# Gestural and Prosodic Development Act as Sister Systems and Jointly Pave the Way for Children’s Sociopragmatic Development

**DOI:** 10.3389/fpsyg.2019.01259

**Published:** 2019-06-12

**Authors:** Iris Hübscher, Pilar Prieto

**Affiliations:** ^1^URPP Language and Space, University of Zurich, Zurich, Switzerland; ^2^Institució Catalana de Recerca i Estudis Avançats, Barcelona, Spain; ^3^Facultat de Traducció i Ciències del Llenguatge, Universitat Pompeu Fabra, Barcelona, Spain

**Keywords:** gesture acquisition, prosody acquisition, sociopragmatic development, multimodal communication and learning, child language acquisition

## Abstract

Children might combine gesture and prosody to express a pragmatic meaning such as a request, information focus, uncertainty or politeness, before they can convey these meanings in speech. However, little is known about the developmental trajectories of gestural and prosodic patterns and how they relate to a child’s growing understanding and propositional use of these sociopragmatic meanings. Do gesture and prosody act as sister systems in pragmatic development? Do children acquire these components of language before they are able to express themselves through spoken language, thus acting as forerunners in children’s pragmatic development? This review article assesses empirical evidence that demonstrates that gesture and prosody act as intimately related systems and, importantly, pave the way for pragmatic acquisition at different developmental stages. The review goes on to explore how the integration of gesture and prosody with semantics and syntax can impact language acquisition and how multimodal interventions can be used effectively in educational settings. Our review findings support the importance of simultaneously assessing both the prosodic and the gestural components of language in the fields of language development, language learning, and language intervention.

## Introduction

Human face-to-face interaction is essentially multimodal and constitutes an organized combination of gesture and speech patterns that jointly convey relevant meanings. For example, speakers typically express focus in discourse through the use of prosodic prominence together with gestural beat movements (e.g., hand, arm, and head movements), which are temporally associated with the focused constituent. Researchers agree that gestures and speech form an integrated system from both a phonological and a semantic standpoint (McNeill, 1992; Goldin-Meadow, 1998; Graziano and Gullberg, 2018). Experimental work largely supports the gesture–speech integration hypothesis in both language production and language comprehension. For example, Kelly et al. (2010) showed how congruent combinations of iconic gestures and speech, expressed in lexical items (for example, saying “chop” while making a chopping hand gesture), as opposed to incongruent combinations of gesture and speech, are integrated through mutual and necessarily bidirectional interactions during language comprehension.

In the last few decades, prosody has been shown to play a pivotal role within the gesture–speech integration system. First, from a temporal point of view, crosslinguistic investigations have shown that the prominent portions of co-speech gestures (e.g., gesture strokes and gesture apexes) tend to temporally align with prosodically prominent positions in speech, (e.g., pitch-accented syllables and peaks of rising pitch accents) (see De Ruiter, 1998 for Dutch; Jannedy and Mendoza-Denton, 2005; Rochet-Capellan et al., 2008 for Brazilian Portuguese; Esposito et al., 2007 for Italian; Ferré, 2011; Esteve-Gibert and Prieto, 2013 for French; Leonard and Cummins, 2011; Loehr, 2012 for English; Ambrazaitis and House, 2017 for French; Esteve-Gibert et al., 2017 for Catalan). In addition, several studies have highlighted the fact that prominent positions, as well as prosodic boundaries, shape gestural coordination patterns and speech planning processes, revealing that prosodic structure acts as an anchoring structure for gesture movements (Esteve-Gibert and Prieto, 2013; Krivokapiæ, 2014; Esteve-Gibert et al., 2017; Graziano and Gullberg, 2018).

Several studies have demonstrated the independent roles of prosody and gesture as sociopragmatic markers (see Kendon, 2004; Kita, 2009; Prieto, 2015 for the role of prosody in pragmatic marking). When looking at the field of pragmatics and pragmatic development more specifically, it becomes clear that the field has been dominated by the analysis of verbal expression. The standard definition of pragmatics refers to the ability to use propositional language in a given context. However, it has been suggested that prosody and non-verbal expressions, such as gestures, also convey meaning in a given context (Bara, 2010). The Bara definition views this type of cue as being something outside of language. This review is based on the assumption that verbal language is only one aspect of communication; that communication is multimodal and includes both visual and vocal expression (e.g., Perniss, 2018).

Recent work has shown that not only do both prosody and gesture convey pragmatic meaning, but, crucially, that they can also be regarded as two sides of the same coin in the conveyance of sociopragmatic meaning (see Krahmer and Swerts, 2005, 2009 for an overview of the audiovisual prosody framework). Brown and Prieto (unpublished) contend that prosody and gesture work in parallel to jointly encode a set of sociopragmatic meanings related to information structure, speech act information, epistemic stance, or politeness. Speakers tend to mark focused elements through prosodic prominence markers such as pitch accentuation, along with head nods, manual beat gestures and eyebrow movements (Dohen and Loevenbruck, 2009 for French; Kim et al., 2014 in English). The presence of these visual features enhances the perception of contrastive focus (e.g., Krahmer and Swerts, 2007; Prieto et al., 2015). Epistemic stance meanings, such as uncertainty or ignorance, are also expressed in both the gestural and prosodic domains: speakers express uncertainty by using facial cues involving eyebrow raising and furrowing, ‘funny faces’ and by means of prosodic cues such as fillers, delays, and rising intonational pitch contours (Krahmer and Swerts, 2005; Dijkstra et al., 2006; Roseano et al., 2016). Similarly, speakers tend to signal politeness by using prosodic cues (e.g., by using a slower speech rhythm and a quieter and lower-pitched voice), together with more mitigated gestures and slower gestural movements (Winter and Grawunder, 2012; Brown et al., 2014; Hübscher et al., 2017a; Brown and Winter, 2018). All of the above supports the hypothesis of Bolinger (1986, p. 199), who suggested that “gesture and speech, specifically intonation, are a single form in two guises, one visible and the other audible,” and argued that gesture and intonation were closely related parts of a single gestural complex that stemmed jointly from the same semantic intent.

Despite the importance of gesture and prosody in communication, research on children’s pragmatic development, has largely focused on the acquisition of lexical and morphosyntactic structures. Language acquisition research has noted the precursor role of prosody and gesture, but chiefly when considering early stages of language development. While prosodic features have been regarded as bootstrapping features for early speech segmentation, word learning, and syntactic development (see Cavalho et al., 2018, for a review), gestures (and specifically deictic gestures, e.g., pointing) have been shown to be strong predictors of early lexical and syntactic acquisition (Capirci et al., 1996; Iverson and Goldin-Meadow, 2005; Özçalişkan and Goldin-Meadow, 2005). However, there is sufficient evidence to hypothesize that prosodic and gestural patterns might be very important components of sociopragmatic development. First, Infant-Directed Speech (IDS) tightly integrates the prosodic and gestural properties of language, which in turn are profitably used by children as they learn to speak. When using IDS, caregivers often exaggerate both the prosodic features of their speech (higher mean pitch, expanded pitch contours, slower speech rate, and longer pauses) and their gestural behavior (more pronounced head and hand movements, more exaggerated facial expressions) in a manner analogous to the acoustic enhancement that occurs in their speech (e.g., see Smith and Strader, 2014). Second, recent results show that infants are extremely sensitive to the temporal synchrony between gestural movements and prosodic patterns. For example, using materials with congruent and incongruent prominence matching patterns, Esteve-Gibert et al. (2015), showed how 9-month-old infants are able to reliably detect whether a manual deictic gesture is aligned with the corresponding metrical patterns (see also Kitamura et al., 2014 for an experiment showing how 8-month-old infants successfully detect matching visual and prosodic displays of continuous IDS speech). Third, from a production point of view, at the onset of word production, infants temporally coordinate their metrically prominent syllables with pointing gestures in an adult-like way (Butcher and Goldin-Meadow, 2000; Esteve-Gibert and Prieto, 2014). Finally, Igualada et al. (2015) showed that the first uses of temporally synchronous pointing-speech combinations by 12-month-old infants have a high predictive value for language (vocabulary) and syntactic outcomes at 18 months (see also Murillo et al., 2018). Given that there is already evidence that gesture and prosody jointly constitute precursor systems for lexical acquisition and syntactic/semantic development (i.e., moving from one to two-word stage), this evidence points to the need to assess the possibility that gesture and prosody also jointly constitute a precursor system for pragmatic development.

Though little is known about the developmental path followed by prosodic and gestural patterns in relation to language acquisition, and specifically in relation to pragmatic development, some researchers have highlighted the need to study gestural and prosodic patterns in tandem with development. Recently, Snow (2017) proposed that intonation and gesture are sister systems marking the same pragmatic functions by focusing on the analysis of protodeclarative sentences in 8- to 16-month-old infants. He claimed that the child’s use of pitch prominence within an utterance vocally designates or “points to” the information focus of the utterance, similarly to a pointing gesture. Very recently, Esteve-Gibert and Guellaï (2018) reviewed a set of developmental studies showing that gestures are tightly linked to prosody at the temporal and the pragmatic levels and across different developmental stages. The authors stressed the importance of clarifying the links between gesture and prosody at different stages in language acquisition. While Snow (2017) looked at the development of focus in initial speech acts and Esteve-Gibert and Guellaï (2018) focused on the role of the temporal and functional integration of gesture-prosody, the present review paper will expand on these results and assess in a systematic way recent evidence showing that gesture and prosody, working in parallel, jointly constitute children’s early development of socio-pragmatic meanings.

The main aim of this paper is to focus on the relevance of prosody and gesture, and the links between the two, in the context of children’s sociopragmatic development. The article first reviews evidence on the role of gesture, Section “Gesture as a Precursor and Predictor of Lexical and Syntactic Development,” and prosody, Section “Prosody as a Precursor and Predictor of Lexical and Syntactic Acquisition,” as independent precursors and predictors of lexical and syntactic development in early infancy. Section “Developmental Evidence That Gesture and Prosody Are Precursors of Pragmatic Development” analyzes evidence that points to the precursor role of both prosody and gesture patterns across four separate components of pragmatic meaning, namely speech act distinctions, information structure, epistemic stance, and politeness marking. First, precursor speech act marking and information focus strategies are assessed in infants aged between 12 and 18 months. Second, epistemic and politeness marking strategies are assessed in 3- to 5-year-old preschoolers. Further complex pragmatic meanings such as irony and deceit will not be part of this review, but see Bosco et al. (2013) for a full review of these meanings, highlighting both perception and production and the inclusion of different means of expression. The focus of this investigation lies in the joint analysis of multimodal prosodic and gesture patterns by considering different types of gestures (hand, as well as head, face, and shoulders) along with prosodic features as well as pitch (e.g., intensity, duration, and voice quality).

## Gesture as a Precursor and Predictor of Lexical and Syntactic Development

Gesture has been found to play a significant role in children’s early language and communicative development. According to Tomasello (1995), the start of intentional communication in infants requires two abilities: (a) the ability to distinguish means and goals in their own actions and in their interlocutor’s actions; and (b) the ability to engage in joint attention frames. By 9 months, infants are involved in complex triadic interactions between ego (the baby), alter (e.g., a parent), and an object of attention (e.g., a ball) (Striano and Tomasello, 2001). In this context, gesture, in the form of pointing, along with eye-gaze and vocalization patterns, is a key ingredient in the management of joint attention.

The pointing gesture has been studied the most as it presents the clearest case of declarative reference (i.e., directing an interlocutor’s attention) within a joint attention framework. Pointing gestures are thought to be used in all human societies (Kita, 2003). In development, pointing is first used by 9- to 12-month-old infants well before they are able to produce their first words (e.g., Bates, 1976; Bates et al., 1979; Acredolo and Goodwyn, 1985; Iverson et al., 1994; Özçalişkan and Goldin-Meadow, 2005). While pointing is deictic (i.e., directive) in nature, infants also make use of other arm movements that serve an iconic function. They convey information about the attributes of an object or mimic actions, such as flapping arms to illustrate flying (Bates et al., 1979). Therefore, gesture comes before speech in language development and gives infants a channel to express declarative reference before they are able to do so through lexical means.

While some studies have demonstrated that gesture precedes verbalization, others have shown that the use of pointing gestures can *predict* lexical development (e.g., Capirci et al., 1996; Iverson and Goldin-Meadow, 2005; Özçalişkan and Goldin-Meadow, 2005; Rowe and Goldin-Meadow, 2009; Igualada et al., 2015). For example, Iverson and Goldin-Meadow (2005) showed that the use of gesture predicts the later use of verbal cues, thereby laying the foundation for the appearance of lexical meanings in speech. By observing the gestural and verbal development of three children between the ages of 10 and 24 months at monthly intervals, these authors found that referents that featured initially in children’s gestural repertoire appeared some months later in their spoken vocabulary. For example, if children pointed to a ball at 14 months, the word ball was highly likely to appear in their vocabulary at 18 months.

Similarly, just as deictic gestures act as precursors to children’s initial acquisition of single words, children combining deictic gestures with speech precedes the production of two-word utterances (Capirci et al., 2005; Iverson and Goldin-Meadow, 2005; Özçalişkan and Goldin-Meadow, 2005; Pizzuto and Capobianco, 2005; Goldin-Meadow, 2009). Studies have found that those children who were the first to produce gesture-plus-word combinations conveying two elements in a proposition (e.g., pointing at a chair while saying “sit”) were also those who first produced two-word combinations (“sit chair”). Özçalişkan and Goldin-Meadow (2005) investigated whether the production of supplementary gesture–speech combinations, (e.g., *eat* + point at cookie) at 14, 18, and 22 months, foreshadowed oncoming changes in children’s speech, thus serving as a forerunner of linguistic advances. They found that the number of supplementary gesture–speech combinations produced by the children significantly increased between the ages of 14 and 22 months. Children first produced various constructions in gesture–speech combinations before the same constructions appeared entirely as speech.

Thus, deictic gestures play clear precursor and predictor roles in lexical and syntactic acquisition, particularly in the early stages of language development. However, less is known about the potential role of pointing gestures in the development of pragmatic skills, given that pointing is used to express basic pragmatic functions. It has been shown that, between the ages of 11 and 12 months, infants begin to produce communicative pointing gestures that have a declarative or requestive pragmatic function (Cochet and Vauclair, 2010). Similarly, infants at 12 months of age have been shown to be able to use the shape of the pointing gesture for specific pragmatic intentions, namely to request an object, inform about its presence, or express interest in it (see Kovács et al., 2014; Liszkowski, 2014 for a review; Tomasello et al., 2007). However, little is known about the relationship between the use of these gestures and the acquisition of pragmatics, or about how different shapes of pointing gestures combine with distinct prosodic patterns to express speech act distinctions. This is partly due to the fact that linguistic pragmatics has been regarded as a primarily verbal phenomenon. Moreover, gestural communication does not disappear with the emergence of word production, and even very young children can encode epistemic stance or politeness information through gesture and prosody. In Section “The Development of Speech Act Marking in Infancy,” we present evidence that, acting as sister systems, prosody and gesture function as precursors in the development of children’s comprehension and deployment of speech acts.

## Prosody as a Precursor and Predictor of Lexical and Syntactic Acquisition

Like gestures, prosodic skills have been shown to arise very early in infants’ communicative development and play an important role in early language acquisition. Special attention has been paid to how prosody facilitates the segmentation of early speech into phonemes, syllables, and syntactic structures (see Cavalho et al., 2018 for a review). In their key study, Gleitman and Wanner (1982) proposed that acoustic cues in speech may help infants to detect syntactic boundaries before lexical knowledge is available (see also Morgan and Newport, 1981; Pinker, 1987; Gleitman et al., 1988). This proposal has become known as the *prosodic bootstrapping hypothesis*. Three important elements are highlighted in this hypothesis, namely that (1) syntax is reliably correlated with prosodic properties; (2) infants are sensitive to the acoustic properties of speech; and (3) infants benefit from these cues when they are processing speech.

Importantly, prosody scaffolds not only children’s early segmentation and syntactic abilities but also their lexical acquisition (see Thorson, 2018 for an overview). As we have noted above, IDS is characterized by slower speech rates and exaggerated pitch excursions, which highlight vocalic and consonantal contrasts and thus help children in their construction of phoneme inventories (Kuhl et al., 1991; Werker et al., 2007; Cristià, 2011). It has been found that the use by a caregiver of such slower speaking rates and more emphatic vowel properties help 21-month-olds to better learn and remember new words (Ma et al., 2011). Thorson and Morgan (2014) showed that word learning is enhanced both when words appear at prosodic phrase boundaries and when new words carry a more prominent pitch accent. Thus, the exaggerated prosodic properties of IDS serve to make the relationship between prosodic form and lexical identity uniquely salient to children (Saint-Georges et al., 2013; Thorson, 2018).

Prosody, like gesture, has been found to provide children with acoustic cues that serve to bootstrap their grammatical and lexical development. However, research also suggests that young infants are able to use prosody for early intentional communication and the marking of speech act information well before the one-word stage (Papaeliou and Trevarthen, 2006; Sakkalou and Gattis, 2012; Esteve-Gibert and Prieto, 2013). Papaeliou and Trevarthen (2006) investigated various acoustic and prosodic parameters of 10-month-old English-acquiring children, comparing them playing by themselves with when they were with their mother. The results of their experiment showed that intentional vocalizations directed at their mother were shorter and displayed higher pitch values than the non-intentional vocalizations they produced when alone. In a later study, Esteve-Gibert and Prieto (2013) analyzed a total of 2,701 vocalizations from a longitudinal corpus of four Catalan-babbling infants aged 0;7 to 0;11 months. They found that infants used different prosodic patterns not only to distinguish communicative vocalizations from investigative ones and to express intentionality, but also to mark speech act information. Specifically, they found that requests and expressions of discontent displayed a wider pitch range and longer duration patterns than responses or statements. Sakkalou and Gattis (2012) tested if 14- and 18-month-old British infants would imitate more intentional actions than accidental ones, when the difference between the two actions was based on the prosody of the word accompanying the actions. During the test phase, the experimenter modeled two different actions on the toy. The intentional action was accompanied by a ‘there’ in a purposeful and satisfied tone of voice, and the accidental action was accompanied by ‘woops’ with a dissatisfied and surprised tone of voice. They found that both 14- and 18-month-old infants were able to understand the intention of the experimenter by responding differently to accidental actions than to intentional actions. Then, in a follow-up experiment, they replaced the English words ‘there’ and ‘whoops’ with novel Greek words that were not known to the infants. Here they found that, purely on the basis of prosody, 18-month-old infants, and to a lesser extent, 14-month-old infants, more often imitated intentional actions than accidental ones. This demonstrates that infants are able to understand the pragmatic value of prosody since they are able to link prosodic properties to intentionality. To summarize, these results show that prelexical infants successfully use a set of prosodic patterns to signal intentional speech and speech act information.

Following on from the above review of research into the separate roles of gesture and prosody in early language development, the next section will discuss recent empirical evidence showing how gesture and prosody work together as an integrated system, and specifically act as precursors in children’s pragmatic development.

## Developmental Evidence That Gesture and Prosody Are Precursors of Pragmatic Development

This section reviews empirical evidence on the role of gesture and prosody in early pragmatic development in terms of four pragmatic components, namely speech act marking, information focus, epistemic stance, and politeness. This review is based on extensive literature searches carried out during the past few years, by adopting an interdisciplinary approach on gesture-, prosody-, and pragmatic-development more generally, including studies stemming from the field of linguistics on the one hand and developmental psychological on the other hand or from the intersections of both. Table 1 summarizes the main features of the studies that have taken into account prosody and non-verbal expressive means, i.e., gestures in the same developmental sample. Converging evidence will be shown from the development of the four above-mentioned socio-pragmatic components that prosody and gestures develop in a parallel and complementary fashion, both from a perception and a production point of view, and that they act as precursors of pragmatic development.

**Table 1:** Overview of the studies that have studied the development of different pragmatic functions by including gestural and/or prosodic cues.

Pragmatic function	References	Design	Ages	Gesture cues	Prosodic cues	Production/Perception	Language acquiring
Speech act	Bates et al., 1975	Observational	0;2, 0;6, 1;0	Pointing gestures	–	Production	Italian
Speech act	Cochet and Vauclair, 2010	Observational	0;11 – 3;01	Pointing gestures	–	Production	French
Speech act	Behne et al., 2012	Experimental	1;0	Pointing gestures	–	Production/ Perception	German
Speech act	Prieto et al., 2012	Observational	0;11 – 1;8	–	Intonation contours	Production	Catalan and Spanish
Speech act	Sakkalou and Gattis, 2012	Experimental	1;2 – 1;6	–	Prosodic contours	Perception	English
Speech act	Esteve-Gibert and Prieto, 2012	Observational	0;7 – 0;11	–	Pitch and duration	Production	Catalan
Speech act	Kovács et al., 2014	Experimental	1;2	Pointing gestures	-	Perception	Hungarian
Speech act	Grünloh and Liszkowski, 2015	Experimental	1;2	Pointing gestures	Intonation contour	Production	Dutch
Speech act	Esteve-Gibert et al., 2016	Experimental	0;11 – 1;2	Pointing gestures	intonation, contour, pitch range and mean syllable duration	Production	Catalan
Speech act	Murillo and Capilla, 2016	Observational/ Semi-structured play	0;9 – 1;3	Pointing gestures	Several acoustic parameters	Production	Spanish
Speech act	Frota et al., 2016	Observational	1;0 – 2;4	–	Intonation and duration	Production	Portuguese
Speech act	Aureli et al., 2017	Experimental	1;0, 1;3, 1;6	Pointing gestures	Duration, mean F0, key, final contour	Production	Italian
Speech act	Esteve-Gibert et al., 2017	Experimental	1;0	Pointing gestures	Pitch range and syllable duration	Perception	Dutch
Information focus	Nicoladis et al., 1999	Observational	2;0 – 3;6	Gestures (iconic, deictic, beat)	–	Production	French-English bilingual
Information focus	Ito et al., 2012	Experimental	6;0 – 8;11	–	Pitch accent	Perception	English
Information focus	Mathew et al., 2014	Experimental	5;0 –7;11	Beat gestures	Rythmic marking of speech	Production	English
Information focus	Igualada et al., 2015	Experimental	1;0	Pointing gestures	Pitch accent	Production	Catalan
Information focus	Kurumada and Clark, 2017	Experimental	4;0-4;11	–	Pitch and duration	Perception	English
Information focus	Snow, 2017	Observational/semi-structured play	0;8 – 1;16	Pointing gestures	Intonation	Production	English
Information focus	Murillo et al., 2018	Observational/ Semi-structured play	0;9, 1;0, 1;3, 1;6	Pointing gestures	Vocalizations	Production	Spanish
Information focus	Esteve-Gibert et al., 2019	Experimental	5;0 – 5;11	Head nods	Syllable duration marking	Perception	French
Epistemic stance	Moore et al., 1993	Experimental	3;0 – 6;11	–	Intonation	Perception	English
Epistemic stance	Liszkowski et al., 2008	Experimental	1;0	Pointing gestures	–	Production	Dutch
Epistemic stance	Kim et al., 2016	Experimental	3;0 – 4;11	Head tilt, head shake, shoulder shrug, looking away	–	Production	German
Epistemic stance	Hübscher et al., 2017a	Experimental	3;0 – 5;11	Head tilt, raised eyebrows	Intonation	Perception	Catalan
Epistemic stance	Bartz, 2017	Observational	1;2 – 3;6	Hand flip (PUOH gesture)	–	Production	English
Epistemic stance	Hübscher, 2018	Experimental	3;0 – 5;11	Head tilt and raised eyebrows	Intonation, fillers and vowel lengthening	Production	Catalan
Politeness	Bates, 1976	Experimental	3;0 – 7;11	–	Intonation	Perception and Production	Italian
Politeness	Shochi et al., 2009	Experimental	9;0 – 10;11	Facial cues	Intonation	Perception	Japanese
Politeness	Hübscher et al., 2018	Experimental	3;0-3;11	Facial cues	Intonation	Perception	Catalan
Politeness	Hübscher et al., 2019	Experimental	3;0– 5;11	Body and facial cues	Pitch, duration, intensity, voice quality	Production	Catalan

### The Development of Speech Act Marking in Infancy

Speech acts provide a useful starting point for the analysis of communicative intent, as many researchers regard them as a bridge between the so-called pre-linguistic and early linguistic stages of language development (see Cameron-Faulkner, 2014 for an overview). Speech acts are a key facet of pragmatic development and also serve as the best example for demonstrating the key role of gesture and prosody in children’s pragmatic development. The most detailed analysis of speech act development was carried out by Bates et al. (1975). They focused on the development of two speech acts, (proto)imperatives and (proto)declaratives, and found that both of these speech acts appeared around the age of 10 months in three Italian-speaking children. These speech acts were first expressed gesturally, and then developed into locutionary acts. Building on this study, Tomasello et al. (2007) proposed that pointing was a communicative act, intended to direct an interlocutor’s attention toward an object for one of three reasons: (1) to help the interlocutor with some information that might be of interest to them (*declarative informative pointing*), (2) to share attention with the interlocutor about an object or event (*declarative expressive pointing*), or (3) to request an object from the interlocutor (*imperative pointing*). Focusing on perception, research has shown that, at 12 months, infants understand whether an adult has produced an attention-directing act such as pointing in order to request an object, inform about its presence, or express interest in it (see Behne et al., 2012; Kovács et al., 2014; Liszkowski, 2014 for a review; Tomasello et al., 2007). Similarly, it has been demonstrated that infants aged from 0;7 to 0;11 can also use prosody to signal the specific pragmatic meaning of intentional vocalizations, with requests and expressions of discontent displaying wider pitch excursions and longer durations, and statements and responses displaying narrower pitch ranges and shorter durations (Esteve-Gibert and Prieto, 2012).

As mentioned above, much of the literature addressing early infant communication abilities comprises separate assessments of either gesture (mainly pointing gestures) or speech modalities, and only a few studies investigate this issue in an integrated fashion (Grünloh et al., 2015; Murillo and Capilla, 2016; Aureli et al., 2017; Esteve-Gibert et al., 2017). From a production perspective, these studies have shown that, across languages, the early production of pointing gestures with declarative or requestive functions are accompanied by specific intonational patterns. For instance, the pattern seen by Grünloh and Liszkowski (2015), where 14-month-old Dutch infants produced flat intonations with requestive pointing gestures and rising contours with declarative pointing gestures, seems to be found in children from many different language communities. On the other hand, language-group specific effects have also been identified. For example, Spanish-acquiring infants (15 months) produced flat intonations with declarative pointing and rising contours with requestive pointing (Murillo and Capilla, 2016), similar to Italian-learning infants (also at 15 months) (Aureli et al., 2017). Esteve-Gibert et al. (2016) showed that Catalan child-directed speech contains specific prosody-gesture multimodal combinations expressing distinct speech act information. In their study, nine caregiver-infant dyads played three games designed to elicit pointing acts with either an expressive, imperative, or informative pragmatic meaning. Results showed that in the three pragmatic situations, caregivers used different combinations of pointing gesture features (e.g., hand shape, gesture duration, and the gesture’s lexical affiliate) and prosodic features (intonation contour, pitch range, and mean syllable duration) to express the three different social intentions.

Studying comprehension, Esteve-Gibert et al. (2017) showed that infants are able to infer meaning from multimodal cues, (e.g., pointing gestures alongside prosodic patterns), before they are able to use spoken language forms. In two different experiments, they tested whether 12-month-old infants would be able to link specific prosodic patterns and hand gestures to a speaker’s underlying social intention (declarative, expressive or requestive). While in the first experiment they tested the role of prosody together with lexical and gestural cues, in the second they tested only the role of prosody and gesture. The results of the first experiment showed that the infants were able to use prosody and gesture to understand the intentions behind an attention-directing act, while the results of the second experiment illustrated that even when there was no lexical information, when the cue was controlled across experimental conditions, infants were able to infer a speaker’s social intentions purely on the basis of prosody and manual gestures. This shows that infants are able to understand different speech acts at 12 months, and suggests that prosody and gestural patterns work in parallel to play a role in infants’ early pragmatic development.

Children’s socio-cognitive and lexical skills increase in concert with their use of prosodic features as they grow older. For example, Portuguese, Catalan, and Spanish children between 1 and 2 years of age produce some basic, pragmatically appropriate, intonation contours, which reflect a variety of speech acts such as vocatives, statements, or requests, and express various intentions (Prieto et al., 2012; Frota et al., 2016). Taken together, these findings demonstrate that both gestural and prosodic cues to speech act information develop in an integrated fashion in the early stages of communicative development and well before children can make the same speech act distinctions by verbal means (see Cameron-Faulkner, 2014).

### The Development of Information Focus

Across languages, the use of prosodic prominence has been highlighted as one of the most reliable cues to various features of information structure, in particular the marking of focus. Prosodic prominence is by no means the only such cue, however. Cross-linguistically, focus can also be expressed by means of specific focus morphemes, syntactic movement of constituents, gestural prominence, or combinations of these elements.

Two recent review chapters (Ito, 2018 on focus perception and Chen, 2018 on focus production) on the subject of the acquisition of prosodic focus highlight the fact that research has produced mixed results about the age at which children begin to develop the ability to use prosodic prominence to mark information structure, with values that vary from 3 to 6 years. This variation may be contingent on the sorts of experimental tasks and measures used (Ito, 2018) or the type of focus marking used in a particular language (Chen, 2018). Specifically, Chen (2018) proposes a cross-linguistic theory for the acquisition of prosodic focus according to which, the specific means of marking focus in a particular language will have a predictable impact on the age at which children are able to produce it, and the order in which different types of focus will be acquired.

Ito (2018) points out that the assumption behind many studies about the development of prosodic focus is that the marking of the information structure of words (new, given, contrastive) must await the development of higher-level discourse abilities. However, while it is true that the acquisition of contrastive focus marking comes later in development, and has been separately studied (e.g., Speer and Ito, 2009; Ito et al., 2012; Kurumada and Clark, 2017), we have some evidence suggesting that information focus might be acquired much earlier. Ito (2018, p. 251) states that, “in theory, infants who are learning to detect word boundaries in running speech may be simultaneously developing the ability to compute gross informational weight for parts of speech that bear prosodic prominence.” In fact, recent studies have shown that children benefit from the prosodic and gestural characteristics of IDS for lexical learning (see Thorson, 2018 for a review). As noted above, a series of studies have shown that the use of prosodic prominence expressing focus, in English, facilitates word learning in toddlers under the age of 2 (e.g., Fernald and Mazzie, 1991; Ma et al., 2011; Thorson and Morgan, 2014).

While the abovementioned studies showed that, from a production perspective, infants are sensitive to prosodic prominence cues signaling the focus referent for lexical learning, studies on early intonational development have shown that children younger than 2 years are able to use prosody to mark novel events and emphasis (Esteve-Gibert and Prieto, 2018; Frota and Butler, 2018). Perhaps the earliest evidence of a more emphatic prosodic realization of information focus is that described by Murillo and Capilla (2016). In their study, the authors explored the acoustic properties of vocalizations in the transition to first words, when they were accompanied with pointing gestures. The duration and pitch properties of the vocalizations were found to be expanded and more similar to those of adult speech when produced with pointing gestures with a declarative function.

It is our view that the combination of gesture (pointing) and prosody (most prominent prosody) is initially used by infants to mark information focus. Following Snow (2017), we suggest that intonation and gesture act as sister systems in the signaling of information focus in protodeclarative sentences used by infants between 8 and 16 months. In the regression patterns he detected in language development, Snow (2017, p. 184) found that pitch prominence, like pointing gestures, were used by children to designate or “point to the information focus of the utterance.” He contends that “the comparison between intonation and gesture shows that the two systems differ markedly in form but they have the same pragmatic function.” For example, protodeclaratives and falling intonation both convey the speaker’s intention to make a statement or share an experience about an object of joint attention.

Further evidence comes from the behavior of 12-month-old children in the declarative pointing task used by Igualada et al. (2015). In the task, children produced a pointing gesture, which was temporally synchronous with the pitch accent in the associated word, as a strategy to successfully initiate and maintain joint attention. We interpret the ability of these 12-month-olds to selectively use this multimodal communicative strategy, to engage an adult in joint attention by drawing his or her attention toward an unseen event or object, as an early manifestation of information focus. The feature that marks novelty in speech, and also predicts later lexical and grammatical development, is the close temporal alignment of both prosody and gesture. Igualada et al. (2015) found that the presence of simultaneous pointing-speech combinations, as opposed to speech-only and pointing-only productions, at 12 months old positively correlated with lexical and grammatical development at 18 months. Later in development, the same predictive pattern arises. A recent study investigated the changes in the temporal synchrony between gesture and speech of multimodal communicative behaviors in the crucial transition from babbling to two-word productions (Murillo et al., 2018). It found that, at 15 months, the proportion of overlap between gestural and vocal elements, and the proportion of gesture strokes overlapping with vocalizations, were both related to lexical development 3 months later. Both studies thus provide evidence about the importance of temporal coordination between gesture and speech for early communication and language learning.

A step forward in the early patterns of pointing gesture–speech integration appears later on, at around 4–5 years of age, when children start developing discourse-based strategies. Manual beat gestures (e.g., hand, arm, or head movements typically associated with prosodic prominence) are used by children to focus specific information in discourse (Nicoladis et al., 1999; Capone and McGregor, 2004; Mathew et al., 2014; Esteve-Gibert et al., 2019). Beat gestures accompanying prosodic prominences constitute a clear example of a more refined multimodal gesture–speech unit used as a discourse-integrated focus-marking strategy. Interestingly, several studies have also shown that beat gestures have a beneficial effect on 4-year-old children’s recall and comprehension of narratives (Llanes-Coromina et al., 2018).

Across languages, contrastive focus marking consistently makes a later appearance than information focus marking. Speer and Ito (2009) and Ito et al. (2012) showed that children could only interpret intonation contour signaling contrasts after the age of 6. Kurumada and Clark (2017) showed that children may be sensitive to contrastive focus intonation as early as age 4. Looking at the production of contrastive focus by considering prosody and gesture alongside each other, Esteve-Gibert et al. (2019) found that 5-year-old French children already use head nods to mark contrastive focus and tend to use this strategy well before they apply prosodic strategies (specifically, syllable-duration-marking strategies). Interestingly, studies on a variety of languages, such as German, English, and Mandarin Chinese, using focus particles like *only*, *also*, or *even*, have shown that children acquire these particles by ages 4 or 5 (Crain et al., 1994; Paterson et al., 2003; Notley et al., 2009; Höhle et al., 2016).

The results reviewed in this section suggest that prelexical children already have considerable knowledge of information focus deriving from a combination of prosodic and gestural features. From the point of view of perception, infants benefit from the use of prosodic and gestural prominence features marking focus in IDS for lexical learning. From the point of view of production, at the prelexical stage infants use integrated productions of prosodic and gestural prominence to initiate joint attention scenarios, and further, to signal simple realizations of information focus. These integrated multimodal units can be regarded as precursors of information focus marking because they represent information status at a rudimentary level. At a later stage, multimodal integration of gesture and prosodic prominence at the discourse level is used by older children in their early use of beat gestures to mark discourse novelty and discourse contrast.

### The Development of Epistemic Stance

Prosody and gesture can be strong markers of epistemic stance across languages. Speakers use different prosodic cues such as slower speech rate and higher pitch levels (Krahmer and Swerts, 2005; Roseano et al., 2016), and gestures such as eyebrow furrowing, head tilt, or shoulder shrugging, to indicate ignorance or uncertainty states (Krahmer and Swerts, 2005; Dijkstra et al., 2006).

Twelve-month-old infants already demonstrate sensitivity to other people’s epistemic states, using contextual cues to distinguish between knowledgeable and ignorant partners. In two experiments, Liszkowski et al. (2008) found that 12-month-olds pointed more often to an object whose location was unknown to their adult interlocutor than to an object whose location was known to the adult. However, it is not until the preschool years that children start to fully understand another person’s beliefs or attitudes and can verbalize such things themselves. Research on the development of epistemic stance, and more specifically, knowledge state (certainty and uncertainty), has focused on the acquisition of lexical and verbal epistemic markers (see Matsui, 2014 for an overview over linguistic expressions of certainty and evidentiality). To situate the age frame for acquisition of lexical and grammatical epistemic markers in relation to perception, it has been found that English-speaking children are able to understand the contrast between *I know* and *I think* by age 4 (Moore et al., 1989). At a similar age they also understand the contrast between *must* and *might* (Moore et al., 1990). Similar results have been found for Korean (Choi, 1995; Papafragou et al., 2007), Cantonese (Lee and Law, 2001; Tardif et al., 2004), Turkish and Puerto Rican Spanish (Shatz et al., 2003), Japanese (Matsui et al., 2006), and Japanese and German (Matsui et al., 2009). Even earlier comprehension of speaker uncertainty has been found in Japanese, where at age 3 children were already able to detect a speaker’s uncertainty encoded through grammatical particles (*yo* = direct evidence vs. *tte* = heresay), compared to a later acquisition of verbal forms that served the same function (Matsui et al., 2006).

Drawing accurate conclusions about another speaker’s knowledge is quite a difficult task that requires both conceptual and linguistic maturity. One early study by Moore et al. (1993) compared children’s (3–6 years old) comprehension of mental state lexicon to their comprehension of mental state prosody. Children had to listen to contrasting pairs of statements by two puppets and guess the location of a hidden object. Each statement pair either differed with respect to the mental state verbs, e.g., *know* vs. *think* or *think* vs. *guess*, or with respect to terminal pitch contour (e.g., falling or rising). While 3-year-olds were not able to use either lexicon or prosody to detect where the object was, 4-year-olds started to do so significantly better in the *know* vs. *think* and falling vs. rising pitch contrast conditions. Furthermore, the *think* vs. *guess* condition was much harder, even for the 5-year-old children, than the *know* vs. *think* condition. Testing whether gestural and prosodic cues might facilitate this task for children, a recent experimental study investigated 3- to 5-year-old Catalan-speaking children’s understanding of a speaker’s uncertainty on the basis of lexical cues (*potser* ‘maybe’), intonational cues (rising intonation), or gestural cues (head tilt and raised eyebrows) (Hübscher et al., 2017b). In a between-subjects design, the children were either exposed to the lexical condition (where they received lexical and gestural cues to uncertainty) or the intonation condition (where they were exposed to intonational and gestural cues to uncertainty). Within each condition, three different presentation formats were used (audio-only, visual-only and audiovisual) as within-subject variables. The results showed that the 3-year-olds performed significantly above chance level when detecting a speaker’s uncertainty on the basis of facial gestural cues, and also performed significantly better when dependent on intonation compared to lexical cues. Similarly, Armstrong et al. (2018) tested 3- to 5-year-old children’s understanding of another type of belief state, a speaker’s incredulity, encoded solely in prosody and gesture, and found that the children performed best when they had access to both cues together, providing similar evidence for a parallel development of prosody and gesture in children’s understanding of another speaker’s belief state.

Several studies have provided evidence that children in their preschool years already display some awareness of their own knowledge state (Pillow and Anderson, 2006; Balcomb and Gerken, 2008; Nilsen et al., 2008; Lyons and Ghetti, 2011, 2013; Paulus et al., 2013). Others have found that children do not acquire this feeling of knowledge before they are 7 (e.g., Beck and Robinson, 2001; Flavell et al., 2001; Pillow and Anderson, 2006). Looking at the production of one’s own knowledge state, it has been detected that English-speaking children at age 3 are able to verbally report on their knowledge state when they are in either total ignorance or total knowledge states, but they struggle to signal partial ignorance until they reach school age (Rohwer et al., 2012). Yet evidence from the analysis of multimodal signaling of ignorance and uncertainty shows that children can do this at an even earlier age. First, with regard to signaling ignorance, a longitudinal study with 64 US-English children who were recorded every 4 months from 14 to 42 months found that at around age 2 the children begin to signal their ignorance through gestural cues such as flipping their palms upward and outward or to the side (Bartz, 2017). At 22 months, one-fifth of the sample had been observed producing such a hand flip to signal ignorance, and by 42 months almost half had done so. Verbal statements of ignorance, such as *I don’t know*, emerged later between 22 and 26 months and then increased rapidly. Furthermore, observing children’s gestural and other non-verbal cues, Kim et al. (2016) showed that when they had only partial access to objects hidden in a box, 4-year-olds displayed their uncertainty by employing a set of uncertainty/ignorance gestures like tilting their head to one side, shaking their head, shrugging their shoulders, or looking away. While they were not yet able to verbally report on their knowledge state, they used gestural cues to mark their uncertain knowledge state. Another study focused on Catalan children’s uncertain stance signaling (Hübscher, 2018). A total of 40 3- to 5-year-old Catalan children participated in a guessing game involving five easy and previously touched and seen objects, and five difficult and not previously seen objects. The results showed that children could already signal uncertainty at age 3 using a set of gestural cues (such as tilted head and raised eyebrows) and prosodic cues (rising intonation, fillers and vowel lengthening), and only later began to employ lexical devices like *maybe* or *I think it is*.

In sum, research has provided converging evidence that preschool-aged children first comprehend and produce knowledge state information, in particular, uncertainty, through gestural and prosodic cues before they do so through appropriate lexical marking. Thus we can view these epistemic multimodal markers as playing a precursory and foundational role during sociopragmatic development.

### The Development of Politeness

There is no consensus in the research about when children’s awareness of politeness emerges (Shatz and Gelman, 1973; Andersen, 1977; Hollos, 1977; James, 1978; Axia and Baroni, 1985; Baroni and Axia, 1989). Based on studies investigating children’s understanding of lexical cues in relation to a speaker’s politeness, Andersen (1977) suggested that English-speaking children start to understand how different lexical politeness markers should be used, depending on the status asymmetry between the speaker and addressee, at around age 4. It has also been found that children’s ability to appropriately use the different lexical and morphosyntactic forms and conventions conveying politeness takes quite some time to develop in a number of languages, including English (e.g., James, 1978; Ervin-Tripp and Gordon, 1986; Sealey, 1999), French (Marcos and Bernicot, 1994; Ryckebusch and Marcos, 2004), Swedish (Aronsson and Thorell, 1999), Norwegian and Hungarian (Hollos and Beeman, 1978) and Greek (Georgalidou, 2008). In Japanese, in which a wide array of politeness attitudes must be acquired, it has been suggested that children’s awareness of the use of the complex politeness forms in gratitude and apology situations begins as early as age 6, but does not approximate that of adults until much later, between ages 13 and 16 (Long, 2010). One of the only and most comprehensive studies with a focus on preschool children’s developing perception (and production) of politeness by including intonation as a cue is Bates’s (1976) study. Bates (1976) experimentally tested whether 60 Italian children aged 3–6 could perceive politeness as encoded in lexical cues or through prosody only. The results showed that the children had acquired the understanding of *per favore*, please, as a politeness marker by age 3, but the use of gentle intonation as a strategy only became significant after 4 years of age.

Some studies have found that facial expressions and affective prosody can act as facilitatory cues in the early detection of politeness by children. One study investigated Japanese 9- and 10-year-olds’ ability to infer a speaker’s politeness vs. impoliteness (Shochi et al., 2009). It found that the presence of facial cues was clearly beneficial for the processing of politeness and impoliteness meanings in this age range. In order to investigate the early comprehension of politeness, and focusing on intonation as a cue, a recent study addressed the question of whether 3-year-old children would be better able to infer a speaker’s polite stance in a request on the basis of intonational cues or facial cues, or through a combination of the two cues (Hübscher et al., 2018). The results showed that children were able to infer significantly above chance, which one of two speakers produced more friendly requests for all three conditions. This demonstrated that children could already detect politeness by exclusively using prosodic or gestural cues at an early age when they were still acquiring the various lexical and morphosyntactic politeness markers.

A recent study investigated children’s production of politeness-related cues in request interactions with varying sociopragmatic factors of cost and social distance (Hübscher, 2018; Hübscher et al., 2019). Surprisingly, already at 3 years of age and increasingly more so between 4 and 5 years, children prosodically adapted their speech in a way similar to adults, when the cost of the request was increased. They also modified their gestural behavior when talking to an adult they did not know, by showing, for example, more raised eyebrows, head tilts, smiles, or slumped shoulders. In general, preschoolers seem able to use both prosody and gesture as mitigating strategies when making requests, either when they imply a high cost or are addressed to a stranger. These results are in line with recent work on the adult expression of politeness indicating a cross-linguistic tendency to use prosodic and gestural mitigating strategies when the interlocutor is of higher status. More specifically, in relation to prosody it has been found that speakers’ polite speech has a slower speech rate, a lower intensity, lower pitch, and also the use of a breathy voice quality (Grawunder and Winter, 2010; Winter and Grawunder, 2012; Hübscher et al., 2017a). Gesture goes in a similar direction. While in English it has been shown that adults display an array of gestural mitigation cues such as raised eyebrows, direct body orientation, and a tense, closed position with small gestures, accompanied by a soft voice (Tree and Manusov, 1998), in Korean speakers use fewer gestures and less physical contact in the polite register, while at the same time using more head nods and bows (Brown et al., 2015).

In short, there is increasing evidence that gesture and prosody not only play a precursor role in children’s pragmatic development during infancy, but also, acting in tandem, continue to play a vital role when children are already able to talk and are acquiring more complex socio-pragmatic skills. This provides clear proof of the integrated nature of gesture and prosody and their shared role as foundational stones in sociopragmatic development.

## Discussion and Conclusion

The present review paper has shown converging evidence from recent research about the pivotal role that prosodic and gesture patterns play in children’s sociopragmatic development across languages by focusing on children’s communicative behaviors between the ages of 1 and 5. In general, the empirical evidence shows that typically developing children achieve pragmatic milestones in gesture and prosody before attaining the same milestones in their use of spoken language forms, and this pattern is broadly consistent across children, languages, and stages of development. While research has traditionally focused on the role of prosody and gesture in the early acquisition of lexical and morphosyntactic elements, fewer studies have concentrated on the ways in which prosody and gesture may be involved in how children learn to express pragmatic meanings. It is those few studies that have been the object of our attention in this review.

First, our review has highlighted the fact that early prosodic and gestural patterns in children seem to overlap in terms of the pragmatic functions for which they are used, which range from speech act and information structure marking to stance-related meanings. There is empirical evidence that the two components are closely linked to each other in crucial phases of development, such that gesture and speech are integrated from a temporal and pragmatic point of view into composite multimodal productions. Children learn to perceive and perform speech acts and 12-month-old infants are able to successfully infer whether an adult’s pointing gestures, together with specific prosodic patterns, convey a declarative, requestive, or expressive social intention (Esteve-Gibert et al., 2017). In many languages, children are also able to produce multimodal combinations of pointing gestures with specific prosodic patterns to express requestive or declarative acts (Grünloh and Liszkowski, 2015; Murillo and Capilla, 2016; Aureli et al., 2017).

Second, regarding the marking of information focus, in accordance with Snow (2017), we have argued that the multimodal integration of pointing gestures and prosodic prominence apparent at 12 months can be considered an expressive precursor of information focus. Moreover, prosodic focus cues present in IDS cues are widely seen to afford children early access to lexical meanings (see Thorson, 2018 for a review) and probably help them to gain access to the form-meaning mapping of lexical pragmatic items acquired later, like epistemic stance and politeness. Two cross-sectional studies showed that 3-year-olds were able to express knowledge state and politeness meanings by modulating prosodic and gestural cues well before they started using verbal strategies (Hübscher, 2018; Hübscher et al., 2019). When expressing politeness, these 3-year-olds used gestural and prosodic cues of mitigation very similar to those employed by adults to express politeness in contexts involving higher social status interlocutors and higher pragmatic costs. Overall, the evidence supports the claim that children at different stages of development are able to identify and use multimodal units that integrate gestural and prosodic features as markers of sociopragmatic meaning.

The evidence reviewed here has also shown that gesture and pragmatic prosody are not only acquired in tandem by children, but also seem to both precede and predict changes in children’s more complex sociopragmatic marking involving lexical forms. Though a few studies have shown that children can begin to express pragmatic meaning by way of gesture alone (e.g., Beaupoil-Hourdel et al., 2015 study illustrating how children undergo a change from expressing negation through embodiment then later move on to symbolic negation, where gesture and speech are completely integrated into composite multimodal productions), recent studies have shown that when gestural and prosodic integration are assessed together, children use this combination of channels to understand and produce a variety of pragmatic meanings before they are able to use spoken language forms involving lexical and morphosyntactic strategies (see sections “The Development of Speech Act Marking in Infancy,” “The Development of Information Focus,” “The Development of Epistemic Stance,” and “The Development of Politeness”).

The precursor role of gesture and prosody should not be surprising. Under normal circumstances children acquire language and develop form-meaning mappings in multimodal settings. It is precisely these multimodal cues that boost children’s early access to meanings and help them gain access to the form-meaning mapping of lexical pragmatic items acquired later (e.g., the usage-based approach to language, Tomasello, 2003). The existing research also confirms the important role of IDS, with its exaggerated prosodic and gestural features, in shaping children’s language learning trajectory.

Even though most of the evidence tends to point to a synchronous development of prosody and gesture, it is also important to try to disentangle the specific trajectories of prosodic and gestural features across developmental stages and languages. In particular, future studies will need to determine whether prosodic features or gestural features, or both, act as independent or integrated precursors of language learning and pragmatic development in relation to the exact path of acquisition at the onset of a particular meaning. Initial results from some recent work on the precursor role of focus prosody and the use of head nods (Esteve-Gibert et al., 2019), seem to indicate that they are independent of each other. Specifically, the results of this study showed that preschoolers (5-year-olds) do not seem to use typical acoustic-prosodic marking in order to distinguish between old, new, and corrective focus information in discourse, at a time when they already use head nod patterns to mark corrective focus. Future research will need to better disentangle the temporal pathway of the acquisition of prosodic and gestural cues encoding different pragmatic meanings.

In general, our review of previous work has revealed the need for a more integrated and detailed multimodal approach to the study of prosody and gesture within the context of language acquisition. First, very few studies have assessed prosody and non-verbal expressive means, e.g., gestures, on the same developmental sample. Second, while developmental studies on gesture and prosody have focused on the strict analysis of manual gestures and intonational pitch patterns (see also Snow, 2017), recent findings have shown the relevance of analyzing non-manual gestures such as head, face, or shoulder movements and prosodic features other than pitch, like intensity, duration, and voice quality. Future work will need to assess, in a more systematic fashion, the overlapping versus the distinctive functions of gesture and prosody with respect to pragmatic encoding; that is, the degree of systematicity of the prosodic and gestural cues as signals of different pragmatic functions, as well as their potential degree of overlap from a functional point of view. Previous studies have found that often different articulators are jointly involved in the marking of one specific pragmatic function, as it is the case, for example, with a speaker’s ignorance: a speaker can use a range of different articulator movements such as head shake, raised eyebrows, lips down, open hand palm up gestures and raised shoulders) to signal unknowingness (see also Brown and Prieto, unpublished). Some of these articulators can also be involved in the marking of other pragmatic meanings. For example, when marking focus, speakers typically raise their eyebrows too. Kendon (2002) analyzed a large number of ‘head shakes’ in naturally occurring interactions in Italian and reported eight different kinds of uses for the head shake, with different pragmatic uses. Even though in the context of language acquisition, most of the studies on the development of different pragmatic functions have investigated individual cues, future research will need to more carefully tease out the development of several and partially overlapping cues used to signal a pragmatic function. Ideally this will be done by taking both an observational and an experimental approach, in such a way that they inform each other’s findings.

More research will also be needed to confirm the value of simultaneous gesture–speech combinations in specific socio-communicative contexts, and their potential predictive value for later language development. There is evidence that early gesture and prosodic integration may be paving the way for future developments in a child’s language skills and that an early command of these multimodal features can have a predictive value in the emergence of later linguistic and grammatical strategies. For example, two studies have shown that the degree to which 12-month-old infants use integrated and simultaneous pointing–speech combinations predicts their later language development. The ability to selectively use this multimodal communicative strategy to successfully initiate and maintain joint attention at 12 months is related to language development at 18 months (Igualada et al., 2015; for similar findings see Murillo et al., 2018).

It would be of interest to gather further evidence from later stages of acquisition such as narrative and discourse development. Although recent evidence points toward the predictor role of iconic gestures (specifically character-viewpoint gestures) used by 5-year-olds for narrative structure at later ages (Demir et al., 2014), little is known about the joint role of both gesture and prosody in the process of narrative development. However, recent work strongly focuses on gestures, as seen in the recent book by McNeill on the analysis of cohesion and clause-linking devices in spontaneous narratives produced by children aged 2 and 3, which traces the changing relationships between speech and gesture in this type of discourse (McNeill, 2016). In our view, in order to gain a complete understanding of the steps by which children acquire language, researchers need to take a multimodal perspective by assessing how the relationship between the four components of gesture, prosody, lexicon, and syntax changes over time. Finally, there is clearly also a need to include more typologically different languages in the analysis in order to assess if and to what degree the relationship between prosody, gesture and other verbal elements of language is shaped by different linguistic systems.

Research has provided evidence that pragmatic development is a multimodal venture in which semantically integrated gesture-prosody units act as foundational elements. Given this, it is not surprising that multimodal language training is being used successfully in language intervention settings. However, these interventions have been developed by separately addressing the gestural and prosodic aspects of language. On the one hand, a set of studies has shown that interventions that teach gesture and verbal imitation skills to young children with Autism Spectrum Disorder (ASD) lead to greater gains in the rate of language use (Ingersoll and Schreibman, 2006; Ingersoll et al., 2007; Ingersoll, 2008). On the other hand, speech therapists have employed interventions that mainly use prosodic features for treatment. One example is Melodic-Based Communication Therapy, which has been found to improve expressive vocabulary, verbal imitative abilities, and pragmatic skills in children with ASD by combining standard melodies and positive reinforcement (Sandiford et al., 2013). Another example, in this case intended to treat patients with non-fluent aphasia, is Melodic Intonation Therapy (MIT), a technique that uses melody and rhythm to improve the patient’s expressive language (Norton et al., 2009). Future research in applied developmental treatments would benefit from more holistic approaches that integrate approaches to language learning by including key components of pragmatic development, specifically prosody and gesture. Interestingly, a recent study assessing gesture- and prosody-based narrative training through beat gestures revealed that training 5-year-old children with the observation of beat gestures boosted their narrative performances (Vilà-Giménez et al., 2019). By the same token, an integrated assessment of these components should be included in present standard assessments of children’s pragmatic skills (Phelps-Terasaki and Phelps-Gunn, 2007; Wiig et al., 2013; Carrow-Woolfolk, 2017).

In summary, the evidence presented in this article has shown that children’s development of pragmatic skills is inherently multimodal, and that the multimodal features of language pave the way for children’s sociopragmatic development later in life. We thus argue for the need to incorporate a multimodal view of language into the field of language acquisition. Prosody and gesture are crucial pieces of the foundational puzzle that children have to solve when developing (socio)pragmatic skills, and it is therefore indispensable that they are included in any enquiry in this field. Although we are still far from having a comprehensive picture of the childhood development of prosody and gesture in communication, a more holistic approach, where the different functions and interactions of prosody and gesture are studied in concert with one another, is sure to enrich the field.

## Author Contributions

All authors listed have made a substantial, direct and intellectual contribution to the work, and approved it for publication.

## Conflict of Interest Statement

The authors declare that the research was conducted in the absence of any commercial or financial relationships that could be construed as a potential conflict of interest.
